# En face image-based classification of diabetic macular edema using swept source optical coherence tomography

**DOI:** 10.1038/s41598-021-87440-3

**Published:** 2021-04-07

**Authors:** Atsushi Fujiwara, Yuki Kanzaki, Shuhei Kimura, Mio Hosokawa, Yusuke Shiode, Shinichiro Doi, Kosuke Takahashi, Ryo Matoba, Yuki Morizane

**Affiliations:** 1 Department of Ophthalmology, Okayama University Graduate School of Medicine, Dentistry and Pharmaceutical Sciences, 2-5-1 Shikata-cho, Kita-ku, Okayama City, Okayama 700-8558 Japan; 2 Department of Orthoptics, Faculty of Rehabilitation, Kawasaki University of Medical Welfare, Okayama, 701-0193 Japan

**Keywords:** Neurological disorders, Metabolic disorders, Medical research

## Abstract

This retrospective study was performed to classify diabetic macular edema (DME) based on the localization and area of the fluid and to investigate the relationship of the classification with visual acuity (VA). The fluid was visualized using en face optical coherence tomography (OCT) images constructed using swept-source OCT. A total of 128 eyes with DME were included. The retina was segmented into: Segment 1, mainly comprising the inner nuclear layer and outer plexiform layer, including Henle’s fiber layer; and Segment 2, mainly comprising the outer nuclear layer. DME was classified as: foveal cystoid space at Segment 1 and no fluid at Segment 2 (n = 24), parafoveal cystoid space at Segment 1 and no fluid at Segment 2 (n = 25), parafoveal cystoid space at Segment 1 and diffuse fluid at Segment 2 (n = 16), diffuse fluid at both segments (n = 37), and diffuse fluid at both segments with subretinal fluid (n = 26). Eyes with diffuse fluid at Segment 2 showed significantly poorer VA, higher ellipsoid zone disruption rates, and greater central subfield thickness than did those without fluid at Segment 2 (*P* < 0.001 for all). These results indicate the importance of the localization and area of the fluid for VA in DME.

## Introduction

Diabetic macular edema (DME) is the most common cause of visual loss in patients with diabetic retinopathy (DR)^1–3^. It is characterized by fluid accumulation and retinal thickening and can occur at any stage of DR. Several treatment modalities for DME, such as retinal photocoagulation, administration of anti-vascular endothelial growth factor (anti-VEGF) agents and steroids, and vitrectomy, are used in clinical practice; therefore, assessment of pathological retinal alterations and DME progression is indispensable for the selection of treatments and their timing.

To date, various classifications have been used to assess disease progression in patients with DME. On the basis of fundoscopy, DME was exclusively classified as focal or clinically significant depending on its distance from the fovea^4,5^. Subsequently, the advent of fluorescein angiography (FA) and optical coherence tomography (OCT) allowed detailed characterization of DME^6–10^. In recent years, classifications based on the pattern of retinal thickening on B-scan images have been used, and these include diffuse retinal thickening, cystoid macular edema, and subretinal fluid (SF)^7,8,11^. However, these classifications are not practical for classifying DME progression because the elucidated patterns are not exclusive of each other and may co-exist with one another. It has also been reported that as DME progresses, the localization of the fluid expands from the inner retinal layer to the outer retinal layer, and the area of the fluid in each retinal layer increases^3,12,13^. However, it is difficult to evaluate the localization and area of the fluid simultaneously on B-scan images because the information obtained from the limited number of retinal cross-sectional images is not sufficient for such evaluations.

The recent advent of swept-source OCT (SS-OCT), which is more penetrating and has a higher scan speed than does conventional spectral domain OCT, has enabled the acquisition of high-resolution, three-dimensional (3D) images of the retinal structure. By using en face images constructed from the 3D images, the changes in the retinal structure can be visualized at an arbitrary retinal depth from a bird’s-eye view^14–17^. For example, we recently investigated epiretinal membrane (ERM) using en face imaging and revealed significant associations of the distribution of ERM and the depth of retinal folds due to retinal traction by ERM with visual functions^17^.

In the present study, we utilized en face imaging to simultaneously evaluate the localization and area of the fluid in patients with DME and proposed a classification based on the findings. We also examined the relationship of the DME type with visual acuity and the structural features of the retina.

## Results

### Demographic data

In total, 128 eyes of 93 Japanese patients with center-involving DME were included. The mean age of the subjects was 64.0 ± 10.6 years (35–87 years), and there were 38 women (40.8%). The average HbA1c value was 7.6% ± 1.7%.

### Classification of diabetic macular edema using en face imaging

**Figure 1:**
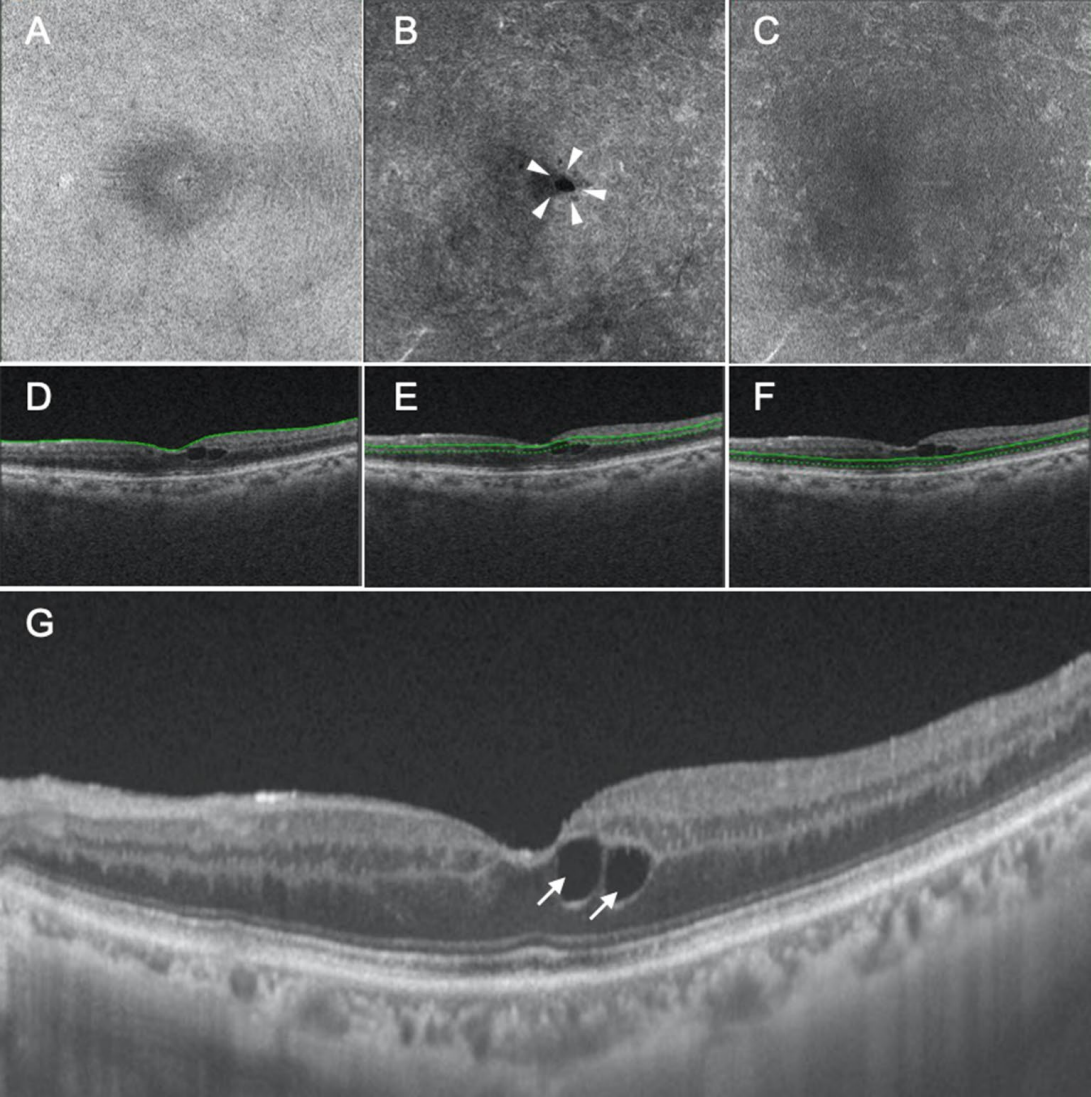
Representative case showing the foveal cystoid space at Segment 1/no fluid at Segment 2 type of diabetic macular edema. (**A**–**C**) En face images of the retinal surface (**A**), Segment 1 (**B**), and Segment 2 (**C**) are presented. The surface of the retina is smooth, and there is no epiretinal membrane (**A**). There are cystoid spaces at Segment 1, with fluid remaining within the fovea (arrowheads, **B**), whereas there is no fluid at Segment 2 (**C**). (**D**–**F**) B-scan images and the green lines show the locations at which the en face images of the retinal surface (**D**), Segment 1 (**E**), and Segment 2 (**F**) were generated. The scan depth, indicated by the distance between the green line and green dotted line, was set to 0 μm for the en face image of the retinal surface (**D**) and 50 μm for the en face images of both Segments 1 and 2 (**E**,**F**, respectively). (**G**) A horizontal B-scan image centered at the fovea is presented. Foveal cystoid spaces are located at Henle’s fiber layer (arrows).

**Figure 2:**
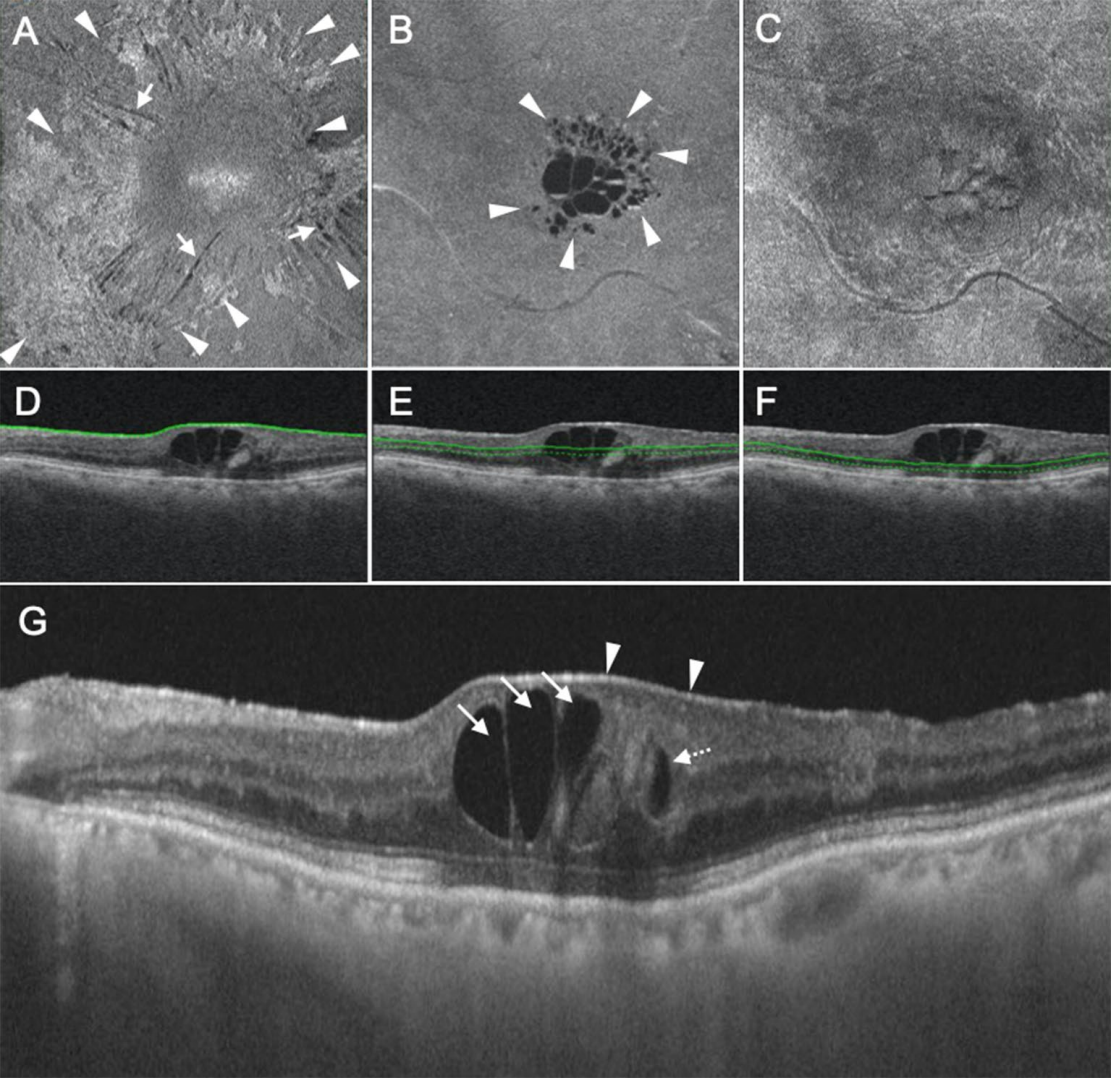
Representative case showing the parafoveal cystoid space at Segment 1/no fluid at Segment 2 type of diabetic macular edema. (**A**–**C**) En face images of the retinal surface (**A**), Segment 1 (**B**), and Segment 2 (**C**) are presented. Epiretinal membrane (arrowheads in **A**) and associated retinal folds (arrows in **A**) can be observed at the surface of the retina (**A**). There are cystoid spaces at Segment 1, with fluid remaining within the parafovea (arrowheads, **B**). There is no fluid at Segment 2 (**C**). (**D**–**F**) B-scan images and the green lines show the locations at which the en face images of the retinal surface (**D**), Segment 1 (**E**), and Segment 2 (**F**) were generated. The scan depth, indicated by the distance between the green line and green dotted line, was set to 0 μm for the en face image of the retinal surface (**D**) and 50 μm for the en face images of both Segments 1 and 2 (**E**,**F**, respectively). (**G**) A horizontal B-scan image centered at the fovea is presented. Parafoveal cystoid spaces are located at both Henle’s fiber layer (arrows) and the inner nuclear layer (dotted arrows). The arrowheads in (**G**) indicate the epiretinal membrane.

**Figure 3:**
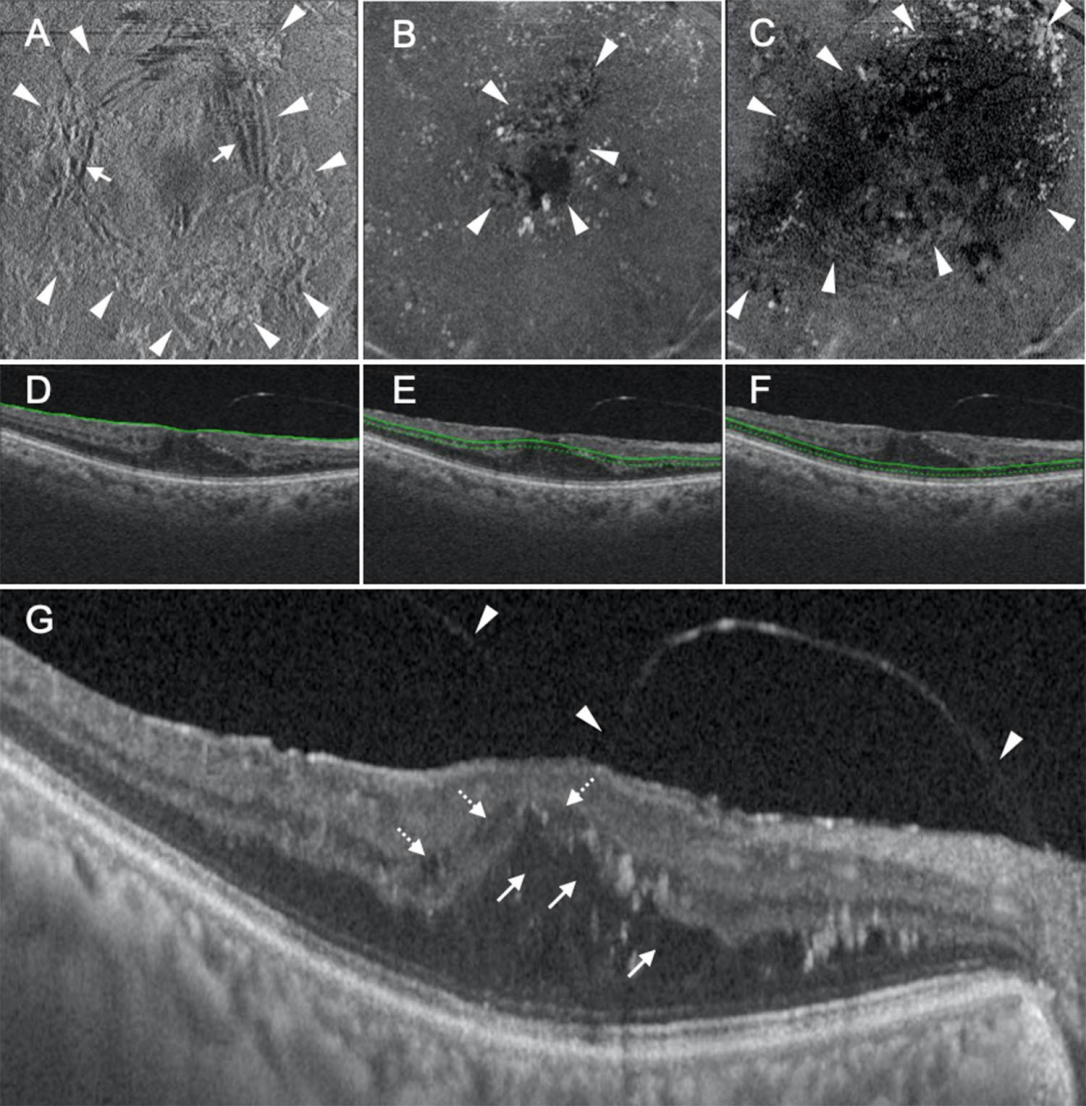
Representative case showing the parafoveal cystoid space at Segment 1/diffuse fluid at Segment 2 type of diabetic macular edema. (**A**–**C**) En face images of the retinal surface (**A**), Segment 1 (**B**), and Segment 2 (**C**) are presented. Epiretinal membrane (arrowheads in A) and associated retinal folds (arrows in A) can be observed at the surface of the retina (**A**). There are cystoid spaces at Segment 1, with the area of fluid remaining within the parafovea (arrowheads in **B**). Segment 2 shows diffuse fluid (arrowheads in **C**). (**D**–**F**) B-scan images and the green lines show the locations at which the en face images of the retinal surface (**D**), Segment 1 (**E**), and Segment 2 (**F**) were generated. The scan depth, indicated by the distance between the green line and green dotted line, was set to 0 μm for the en face image of the retinal surface (**D**) and 50 μm for the en face images of both Segments 1 and 2 (**E**,**F**, respectively). (**G**) A horizontal B-scan image centered at the fovea is presented. There are parafoveal cystoid spaces at both Henle’s fiber layer (arrows in **G**) and the inner nuclear layer (dotted arrows in **G**). The fluid at Segment 2 observed on the en face image is not clearly visualized on this cross-sectional image. The arrowheads in (**G**) show incomplete posterior vitreous detachment.

**Figure 4:**
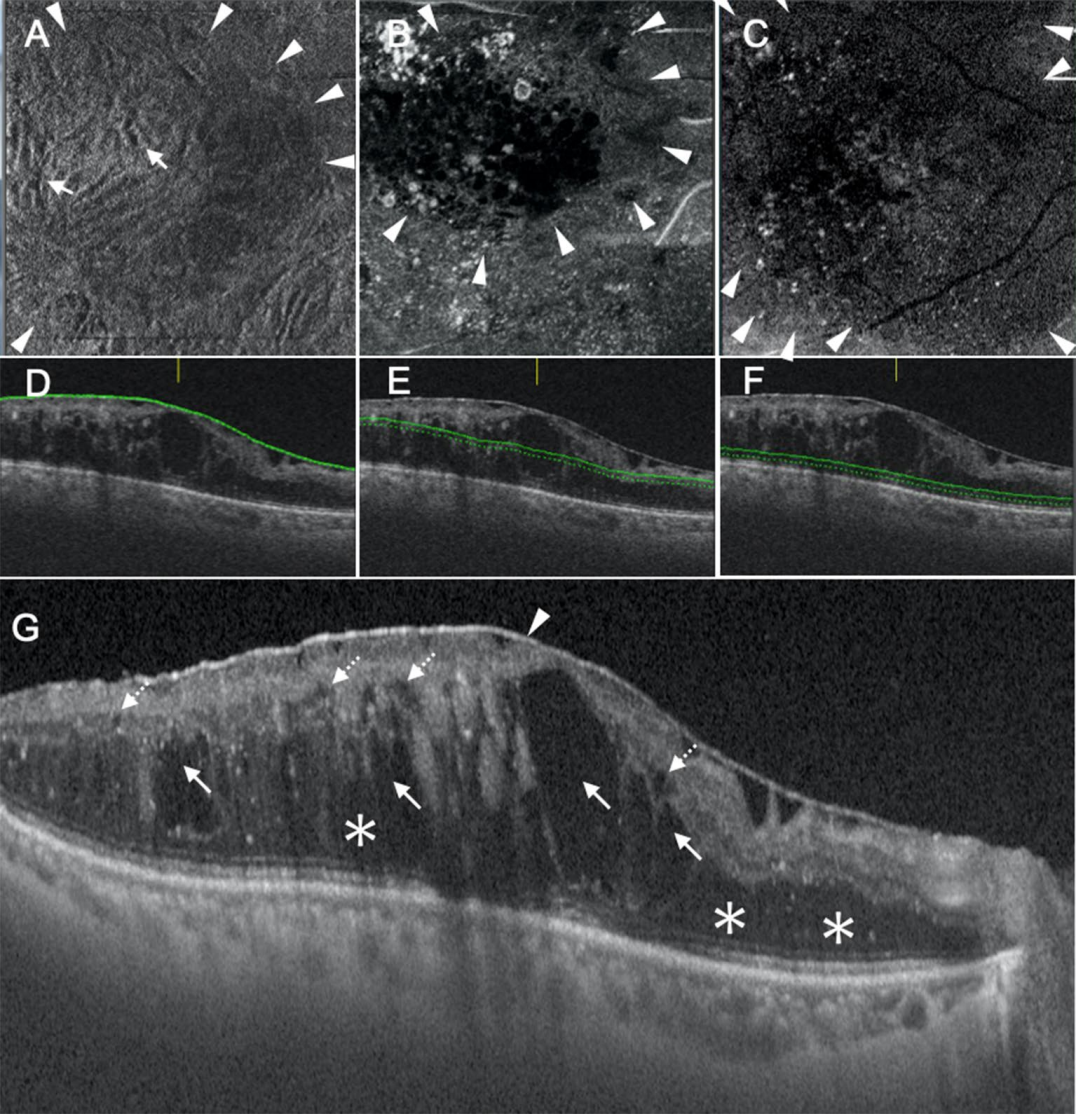
Representative case showing the diffuse fluid at Segment 1/diffuse fluid at Segment 2 type of diabetic macular edema. (**A**–**C**) En face images of the retinal surface (**A**), Segment 1 (**B**), and Segment 2 (**C**) are presented. Epiretinal membrane (ERM; arrowheads in **A**) and associated retinal folds (arrows in **A**) can be observed at the surface of the retina (**A**). There is diffuse fluid with an area larger than the parafoveal area at both Segments 1 and 2 (arrowheads in **B**,**C**). (**D**–**F**) B-scan images and the green lines show the locations at which the en face images of the retinal surface (**D**), Segment 1 (**E**), and Segment 2 (**F**) were generated. The scan depth, indicated by the distance between the green line and green dotted line, was set to 0 μm for the en face image of the retinal surface (**D**) and 50 μm for the en face images of both Segments 1 and 2 (**E**,**F**, respectively). (**G**) A horizontal B-scan image centered at the fovea is presented. The fluid at Segment 1 is located at both Henle’s fiber layer (arrows in **G**) and the inner nuclear layer (dotted arrows in **G**). There is diffuse fluid at Segment 2 (asterisks in **G**). The arrowhead in (**G**) shows ERM.

**Figure 5:**
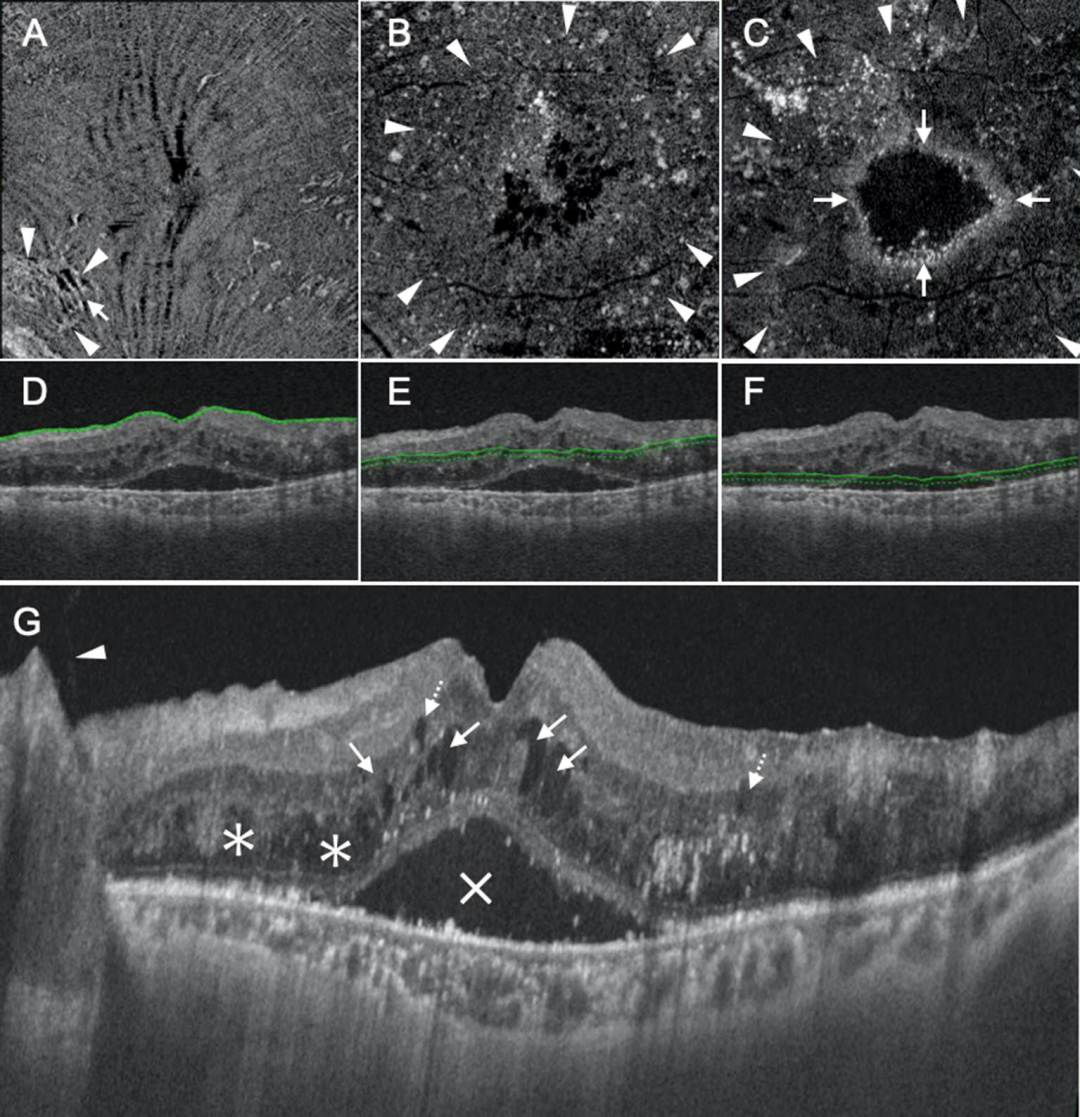
Representative case showing the diffuse fluid at Segment 1/diffuse fluid with subretinal fluid at Segment 2 type of diabetic macular edema. (**A**–**C**) En face images of the retinal surface (**A**), Segment 1 (**B**), and Segment 2 (**C**) are presented. Epiretinal membrane (arrowheads in **A**) and associated retinal folds (arrows in **A**) can be observed at the surface of the retina. There is diffuse fluid with an area larger than the parafoveal area at both Segments 1 and 2 (arrowheads in **B**,**C**). Subretinal fluid, visualized as a high-intensity ring-shaped region, can also be observed at Segment 2 (arrows in **C**). (**D**–**F**) B-scan images and the green lines show the locations at which the en face images of the retinal surface (**D**), Segment 1 (**E**), and Segment 2 (**F**) were generated. The scan depth, indicated by the distance between the green line and green dotted line, was set to 0 μm for the en face image of the retinal surface (**D**) and 50 μm for the en face images of both Segments 1 and 2 (**E**,**F**, respectively). (**G**) A horizontal B-scan image centered at the fovea is presented. The fluid at Segment 1 is located at both Henle’s fiber layer (arrows in **G**) and the inner nuclear layer (dotted arrows in **G**). There is diffuse fluid at Segment 2 (asterisks in **G**). The X in (**G**) indicates subretinal fluid and the arrowhead in (**G**) shows that the posterior hyaloid is detached from the retina completely but remains attached to the optic disc.

We segmented the retina as follows: Segment 1, which mainly comprised the inner nuclear layer (INL) and outer plexiform layer (OPL), including Henle’s fiber layer; and Segment 2, which mainly comprised the outer nuclear layer (ONL). We then classified the findings in the segments as follows: Segment 1, no fluid (NF), foveal cystoid space (FC), parafoveal cystoid space (PC), and diffuse fluid (DF); and Segment 2, NF, DF, SF, and DF with SF. Thus, there were 15 combinations in total (Table 1). Among the 15 types of DME, only five were actually observed (Table 1). The first observed type was characterized by FC at Segment 1 and NF at Segment 2 (FC/NF type), and this group included 24 eyes (18.8%; Table 1; Fig. 1). The fluid was located only at Henle’s fiber layer, thus sparing the other layers, including the INL (Fig. 1). The second observed type was characterized by PC at Segment 1 and NF at Segment 2 (PC/NF type); this group included 25 eyes (19.5%; Table 1; Fig. 2). The fluid was located at both Henle’s fiber layer and the INL (Fig. 2). The third observed type was characterized by PC at Segment 1 and DF at Segment 2 (PC/DF type), and this group included 16 eyes (12.5%; Table 1; Fig. 3). The fourth observed type was characterized by DF at both segments (DF/DF type), and it accounted for 37 eyes (28.9%; Table 1; Fig. 4). The final observed type was characterized by DF at both segments, with SF (DF/DF + SF type), and it was observed in 26 eyes (20.3%; Table 1; Fig. 5). In all eyes with fluid at Segment 2 (PC/DF, DF/DF, and DF/DF + SF), the area of fluid was larger than the parafoveal area.

**Table 1:** Classification of diabetic macular edema based on en face images.

Segment 1				Segment 2				N (%)
No fluid	Foveal cystoid space	Parafoveal cystoid space	Diffuse fluid	No fluid	Diffuse fluid	Subretinal fluid	Diffuse fluid with subretinal fluid	
●					●			0
●						●		0
●							●	0
	●			●				24 (18.8%)
	●				●			0
	●					●		0
	●						●	0
		●		●				25 (19.5%)
		●			●			16 (12.5%)
		●				●		0
		●					●	0
			●	●				0
			●		●			37 (28.9%)
			●			●		0
			●				●	26 (20.3%)

### Relationships of the classification of diabetic macular edema with visual acuity and structural features of the retina

**Figure 6:**
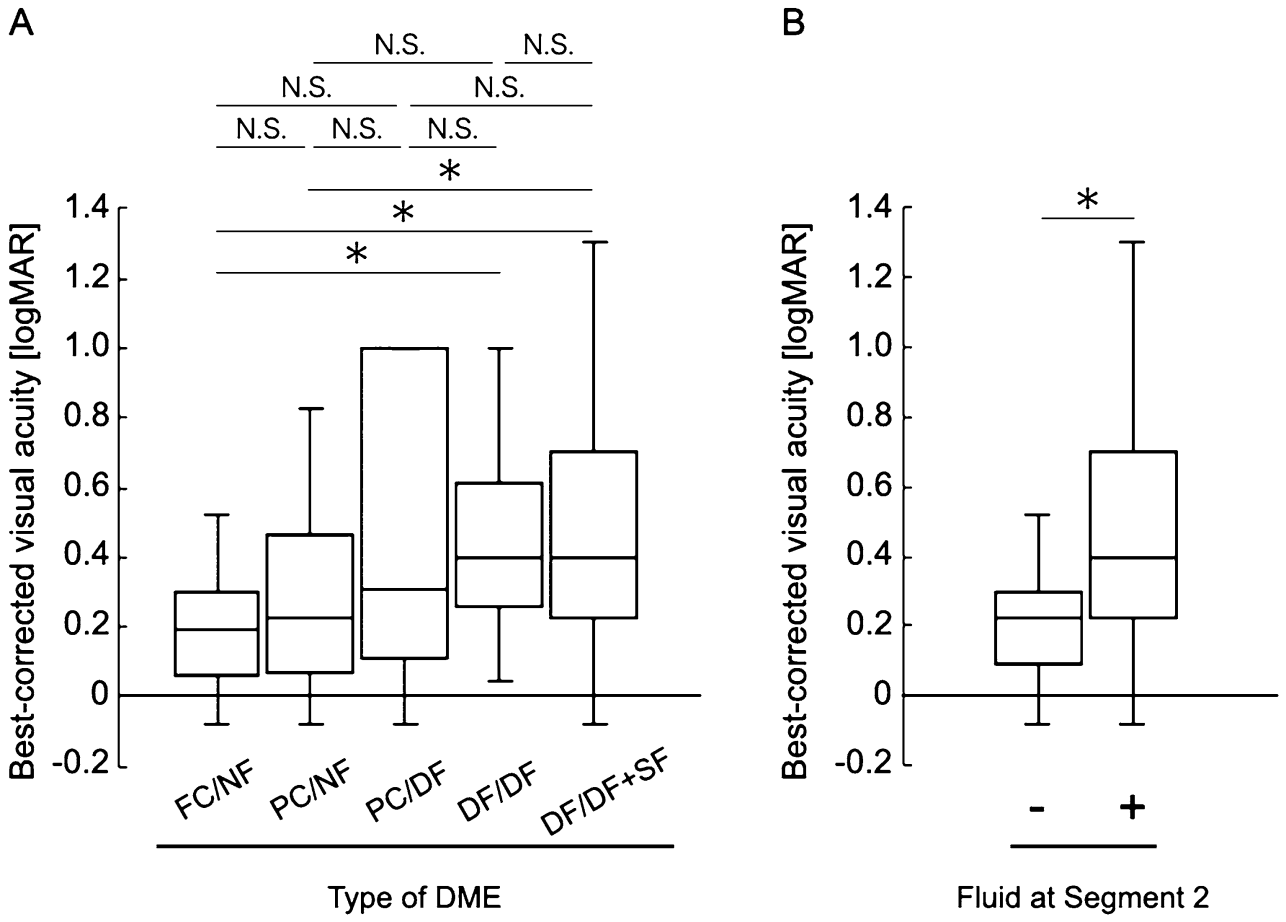
Relationship between visual acuity and the en face image-based classification of diabetic macular edema (DME). (**A**) Best-corrected visual acuity values are compared among the DME types. (**B**) Best-corrected visual acuity values are compared according to the presence or absence of fluid at Segment 2. *DME* diabetic macular edema, *logMAR* logarithm of the minimal angle of resolution, *FC/NF* foveal cystoid space/no fluid type, *PC/NF* parafoveal cystoid space/no fluid type, *PC/DF* parafoveal cystoid space/diffuse fluid type, *DF/DF* diffuse fluid/diffuse fluid type, *DF/DF* + *SF* diffuse fluid/diffuse fluid with subretinal fluid type, *N.S.* not significant; *P < 0.001.

**Figure 7:**
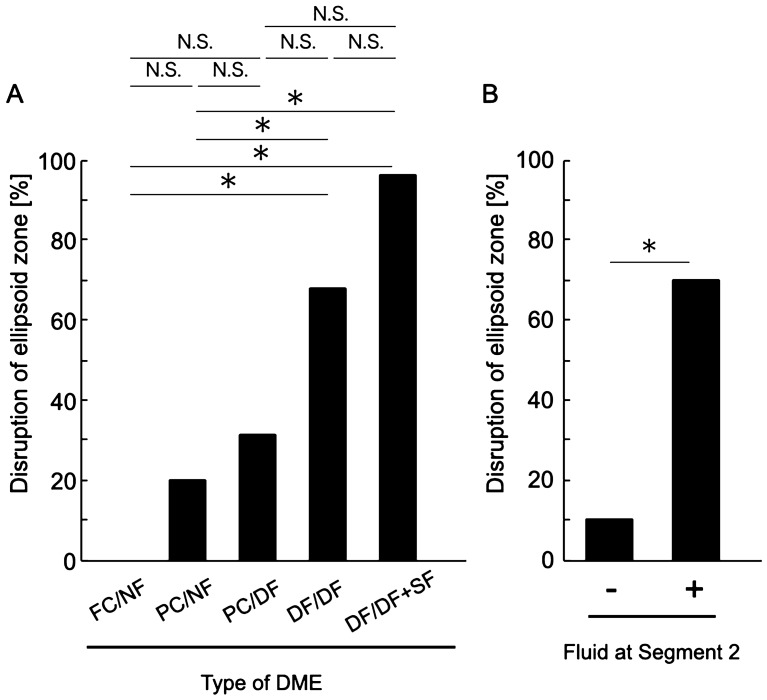
Relationship between ellipsoid zone (EZ) disruption and the en face image-based classification of diabetic macular edema (DME). (**A**) EZ disruption rates are compared among the DME types. (**B**) EZ disruption rates are compared according to the presence or absence of fluid at Segment 2. *DME* diabetic macular edema, *FC/NF* foveal cystoid space/no fluid type, *PC/NF* parafoveal cystoid space/no fluid type, *PC/DF* parafoveal cystoid space/diffuse fluid type, *DF/DF* diffuse fluid/diffuse fluid type, *DF/DF* + *SF* diffuse fluid/diffuse fluid with subretinal fluid type, *N.S.* not significant, *P < 0.001.

**Figure 8:**
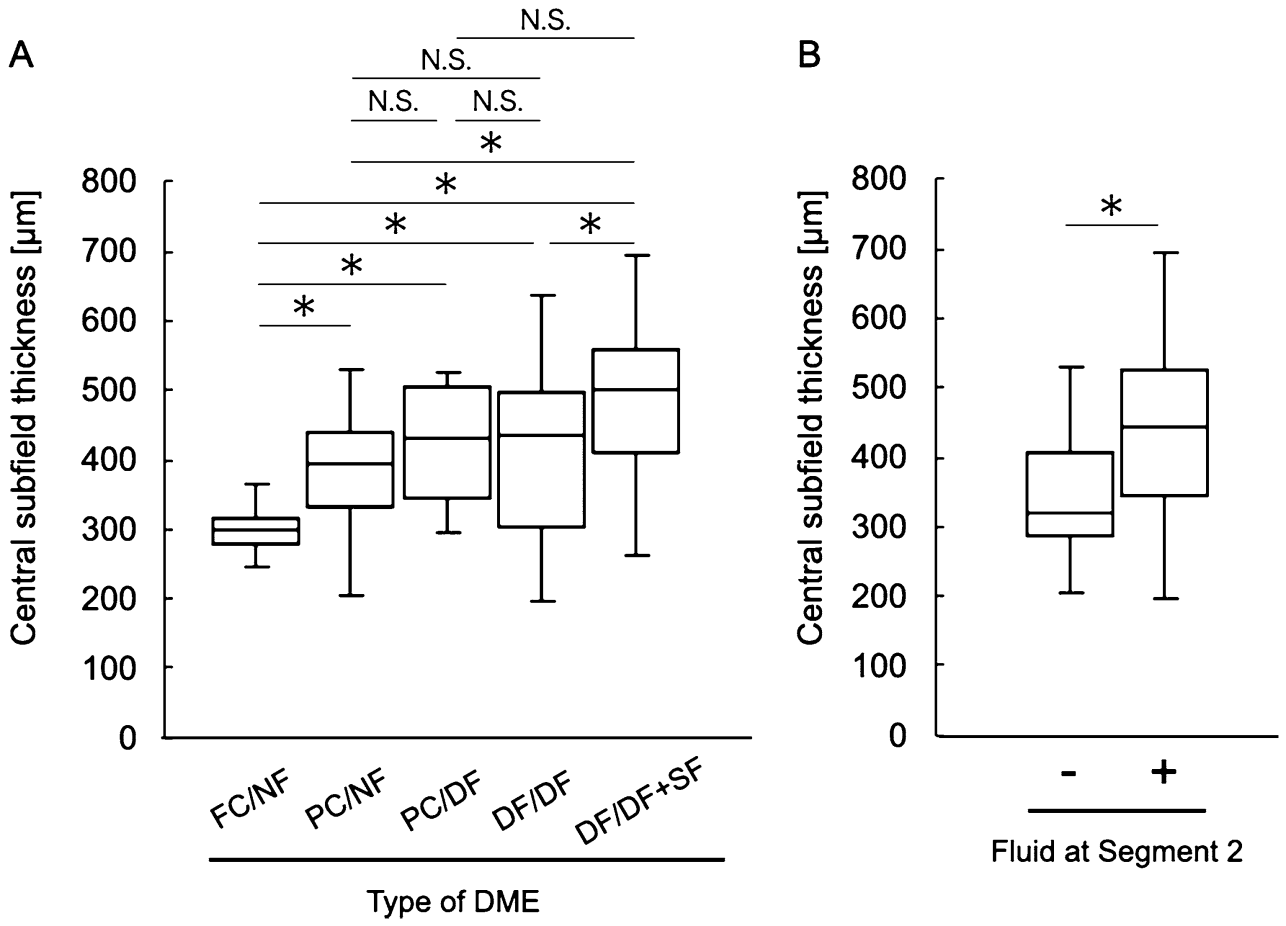
Relationship between the central subfield thickness (CST) and the en face image-based classification of diabetic macular edema (DME). (**A**) CST values are compared among the DME types. (**B**) CST values compared according to the presence or absence of fluid at Segment 2. *DME* diabetic macular edema, *FC/NF* foveal cystoid space/no fluid type, *PC/NF* parafoveal cystoid space/no fluid type, *PC/DF* parafoveal cystoid space/diffuse fluid type, *DF/DF* diffuse fluid/diffuse fluid type, *DF/DF* + *SF* diffuse fluid/diffuse fluid with subretinal fluid type, *N.S.* not significant, *P < 0.001.

**Figure 9:**
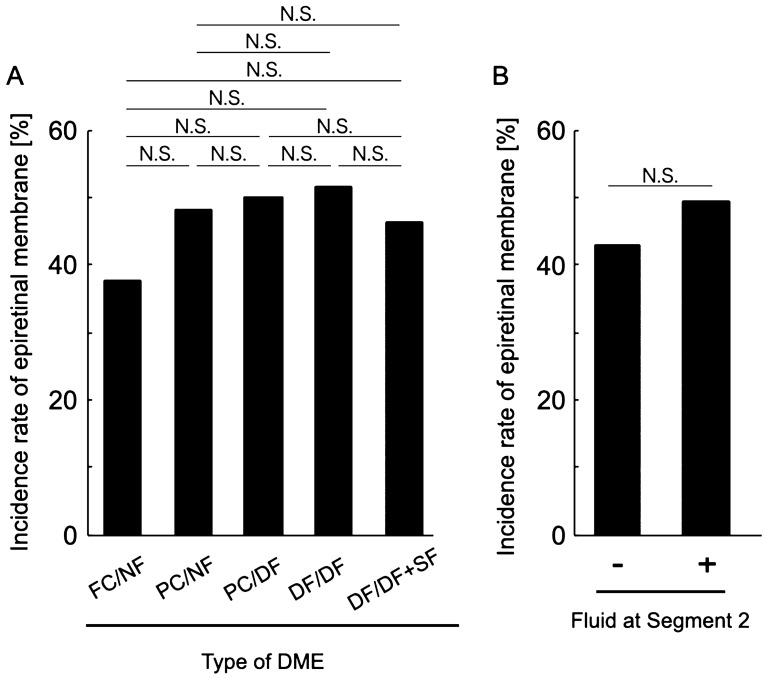
Relationship between the incidence of epiretinal membrane (ERM) and the en face image-based classification of diabetic macular edema (DME). (**A**) The incidence of ERM is compared among the DME types. (**B**) The incidence of ERM is compared according to the presence or absence of fluid at Segment 2. *DME* diabetic macular edema, *FC/NF* foveal cystoid space/no fluid type, *PC/NF* parafoveal cystoid space/no fluid type, *PC/DF* parafoveal cystoid space/diffuse fluid type, *DF/DF* diffuse fluid/diffuse fluid type, *DF/DF* + *SF* diffuse fluid/diffuse fluid with subretinal fluid type, *N.S.* not significant.

Best-corrected visual acuity (BCVA) was significantly different among the different types of DME (*P* = 0.001, Table 2; Fig. 6), whereas there were no significant differences in age, sex, HbA1c level, and the presence of incomplete posterior vitreous detachment (PVD; *P* = 0.062, 0.324, 0.521 and 0.185, respectively; Table 2). There were no significant differences in BCVA among the FC/NF, PC/NF, and PC/DF types and among the PC/DF, DF/DF, and DF/DF + SF types (Fig. 6). When we classified the eyes according to the presence or absence of fluid in Segment 2, the BCVA of eyes with diffuse fluid in Segment 2 (PC/DF, DF/DF, and DF/DF + SF types) was significantly worse than that of eyes without fluid in Segment 2 (FC/NF and PC/NF types; 0.23 ± 0.22 and 0.48 ± 0.34, respectively; *P* < 0.001; Fig. 6). In accordance with the BCVA findings, the ellipsoid zone (EZ) disruption rate was significantly higher for eyes with fluid in Segment 2 (PC/DF, DF/DF, and DF/DF + SF types) than for eyes without fluid in Segment 2 (FC/NF and PC/NF types; 10.2% and 69.6%, respectively; *P* < 0.001; Table 2; Fig. 7), while the central subfield thickness (CST) was significantly greater in the former type (PC/DF, DF/DF, and DF/DF + SF types) than in the latter type (FC/NF and PC/NF types; 345.65 ± 75.83 and 438.69 ± 119.44 μm, respectively; *P* < 0.001; Table 2; Fig. 8). To clarify the factors associated with EZ disruption, we performed logistic regression analysis with the classification of DME, BCVA, and CST as independent variables. The results showed that the classification of DME and the BCVA were significantly associated with EZ disruption (classification of DME: odds ratio, 4.47; 95% confidence interval, 2.57–7.76, *P* < 0.001; BCVA: odds ratio, 72.17; 95% confidence interval, 7.73–673.71; *P* < 0.001). There was no significant difference in the incidence of ERM and maximum depth of retinal folds (MDRF) among the different DME types (*P* = 0.876 and 0.528, respectively; Table 2; Fig. 9).

**Table 2:** Comparisons according to the type of diabetic macular edema determined on the basis of en face images.

Type (segment 1/segment 2)	Foveal cystoid space/no fluid	Parafoveal cystoid space/no fluid	Parafoveal cystoid space/diffuse fluid	Diffuse fluid/diffuse fluid	Diffuse fluid/diffuse fluid with subretinal fluid	*P* value
N (%)	24 (18.8%)	25 (19.5%)	16 (12.5%)	37 (28.9%)	26 (20.3%)	0.067
Age (years)	64.4 ± 12.2	62.3 ± 11.5	70.3 ± 8.3	63.9 ± 8.8	60.2 ± 11.6	0.062
Sex (female/male)	6/18	12/13	8/8	16/21	8/18	0.324
HbA1c (%)	7.2 ± 1.4	6.9 ± 1.0	7.3 ± 1.6	8.2 ± 1.9	8.1 ± 2.1	0. 521
BCVA (LogMAR)	0.17 ± 0.20	0.27 ± 0.26	0.47 ± 0.40	0.46 ± 0.30	0.50 ± 0.35	0.001
Incomplete PVD (%)	10 (41.7%)	8 (32.0%)	7 (43.8%)	16 (43.2%)	17 (65.4%)	0.185
Disruption of ellipsoid zone (%)	0 (0%)	5 (20.0%)	5 (31.3%)	25 (67.6%)	25 (96.2%)	< 0.001
Central subfield thickness (μm)	304.4 ± 42.7	385.2 ± 80.4	420.8 ± 82.4	407.9 ± 129.0	493.5 ± 106.0	< 0.001
Incidence rate of epiretinal membrane (%)	9 (37.5%)	12 (48.0%)	8 (50.0%)	19 (51.4%)	12 (46.2%)	0.876
Maximum depth of retinal folds (μm)	49.7 ± 33.8	41.4 ± 26.0	57.9 ± 16.0	54.3 ± 44.2	57.2 ± 46.5	0.528

*BCVA* best-corrected visual acuity, *LogMAR* logarithm of the minimal angle of resolution, *PVD* posterior vitreous detachment.

## Discussion

In this study, we proposed a classification for DME that was based on en face images constructed using SS-OCT. This en face image-based classification differs from the conventional B-scan image-based classification, which includes diffuse retinal thickening, cystoid macular edema, and SF^7,8,11^, in that DME is not classified only by the pattern of retinal thickening; rather, it is classified by combining two important elements for its progression, namely localization of the fluid (retinal layer at which the fluid exists) and the area of the fluid. To date, the process of DME progression has been primarily studied using autopsied eyes, FA, and B-scan OCT imaging^4,6,8,9,18^. However, in autopsied eyes, which only provide information for a particular section, postmortem changes and changes occurring during the process of specimen fixation need to be considered in order to determine the localization of the fluid^13^. With regard to FA, although the retina can be visualized from a bird’s eye view, and the existence of vascular leakage can also be visualized, it is difficult to determine the precise area of the fluid because the area of fluorescence leakage expands as time elapses during photography. Furthermore, we cannot locate the layer at which the fluid exists or visualize the SF. With regard to B-scan images, the information obtained from the limited number of retinal cross-sectional images is not sufficient for simultaneous evaluation of the location and area of the fluid. Furthermore, when B-scan images are constructed using SD-OCT, the signal intensity is attenuated because of the short wavelength of the light source, and the deep part of the retina cannot be fully visualized in eyes with severe DME. In this study, we utilized en face images constructed using SS-OCT, which has two advantages over conventional SD-OCT. The first advantage is its high scanning speed (100,000 A-scans per second), which enables the capture of 3D images of the retina with high resolution in a short period of time; this allowed us to construct the en face images used in this study^15,19,20^. The second advantage is the long wavelength of the light source (1060 nm); this light shows better tissue penetration and allows for deeper retinal visualization, even in situations where fluid is present in the retina^15,19,20^. On the en face images, the fluid itself was clearly visualized as a well-demarcated, low-intensity region. Furthermore, considering the fact that the inner plexiform layer (IPL) and retinal pigment epithelium (RPE) are hardly disrupted in DME because the layers with fluid accumulation are limited to INL, Henle’s fiber layer, ONL, and the subretinal space^3,12,13^, we found that the division into Segment 1 (mainly from the INL to the OPL, including Henle’s fiber layer) and Segment 2 (mainly from the ONL to the RPE) can be automatically and objectively performed by utilizing the boundary between the IPL and INL (IPL/INL) and RPE as reference planes for flattening the 3D retinal images. As a result, it was possible to separately investigate the area of the fluid at Segments 1 and 2 (Figs. 3, 4, 5). As shown in Fig. 3, even when the fluid at Segment 2 was difficult to detect on B-scan images centered at the fovea, en face images clearly showed the presence of diffuse fluid at Segment 2. Although the fluid at Segment 1 was subtyped with the area of fluid based on the ETDRS map, Segment 2 was only evaluated for the presence of fluid because the area of fluid at Segment 2 was larger than the parafoveal area in all cases.

In the present study, eyes with fluid at Segment 2 showed significantly worse visual acuity than did those without fluid at Segment 2 (Fig. 6). On the other hand, the extent of fluid at Segment 1 did not affect the visual acuity. This result was concordant with the relationship between the DME type and EZ continuity; the EZ disruption rate was significantly higher for eyes with fluid at Segment 2 (Fig. 7). Collectively, these results were in good agreement with the results of studies that used B-scan images and reported that fluid accumulation in the ONL and the subretinal space is the most important pathological change that reduces retinal sensitivity^3,12,21^. It is considered that retinal sensitivity decreases in the presence of fluid at the outer retinal layer because the protein-rich fluid at the outer layer may disturb both oxygenation and elimination of metabolites from the photoreceptor layer^12^. As shown in Figs. 6A, 7A, there was no significant difference in visual acuity and the EZ disruption rate between the DF/DF + SF and DF/DF groups. One of the possible reasons for these results may be the small number of cases showing each type.

In this study, the proposed en face image-based classification of DME was not significantly associated with incomplete PVD, the presence of ERM, and the strength of retinal traction due to ERM. It is important to consider the status of retinal traction, which is mediated by the taut, thickened posterior hyaloid, vitreoretinal adhesion, and ERM, when considering treatment options for DME^22–26^. For eyes in which retinal traction largely accounts for the pathological condition, vitreous surgery should be chosen in order to release the traction because drugs such as anti-VEGF agents and steroids cannot sufficiently resolve the pathological condition^24,27–30^. The incidence of ERM in cases of DME has been reported to be 13–34%^31–34^, which is lower than that observed in the present study (46.9%). One possible explanation for this difference is the lower possibility of missed ERM on en face images than on B-scan images because the former provide a bird’s eye view of the retina.

Unlike that of idiopathic ERM, the pathogenesis of ERM associated with DME is believed to involve chronic inflammation due to hypoxia, oxidative stress, and upregulation of VEGF^1,3^. Chronic inflammation induces and strengthens the adhesion of the posterior vitreous cortex to the internal limiting membrane (ILM) and promotes the proliferation of Müller cells, myofibroblast-like cells, and macrophages, which are the main cellular components of ERM associated with DME^3,35,36^. Accordingly, we hypothesized that, in cases of severe DME, inflammation would increase, ERM would be likely to develop, and the retinal traction due to thickening and contraction of ERM would be likely to increase. Contrary to our expectations, however, the severity of DME was not associated with the incidence of ERM or retinal traction due to ERM, which was estimated from MDRF. Because MDRF for each DME type (from 41.4 ± 26.0 to 57.2 ± 46.5 μm; Table 2) was not large relative to that observed with idiopathic ERM^17^, we speculate that one possible reason for the lack of association between the DME severity and MDRF is that the retina with DME may be less prone to wrinkling than the normal retina on application of traction because its physical properties are different from those of the normal retina because of fluid accumulation. Further investigation with a larger number of cases is warranted.

**Figure 10:**
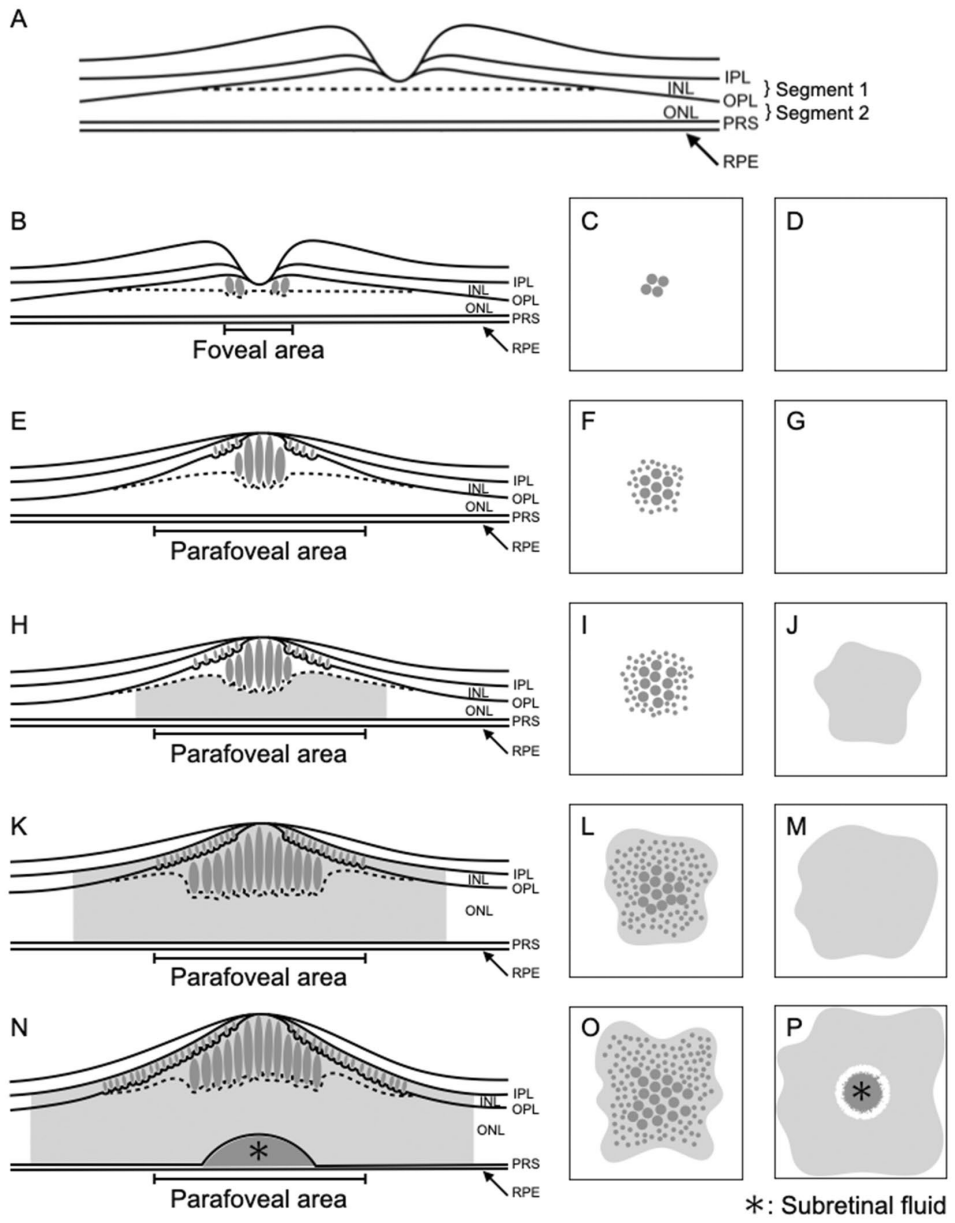
Schematic illustration of the en face image-based classification of diabetic macular edema (DME). (**A**,**B**,**E**,**H**,**K**,**N**) The illustrations indicate the cross-section of the retina. The dotted line indicates the inferior border of Henle’s fiber layer. (**C**,**F**,**I**,**L**,**O**) The illustrations indicate the en face image of Segment 1. (**D**,**G**,**J**,**M**,**P**) The illustrations indicate the en face image of Segment 2. (**A**) This illustration indicates the locations of Segments 1 and 2 in the normal retina. Segment 1 mainly comprises the inner nuclear layer (INL) and outer plexiform layer, including Henle’s fiber layer. Segment 2 mainly comprises the outer nuclear layer. (**B**–**D**) This is the foveal cystoid space/no fluid type. In Segment 1, foveal cystoid spaces can be observed at Henle’s fiber layer (**B**,**C**). There is no fluid at Segment 2 (**B**,**D**). (**E**–**G**) This is the parafoveal cystoid space/no fluid type. In Segment 1, parafoveal cystoid spaces can be observed at both Henle’s fiber layer and INL (E and F). There is no fluid at Segment 2 (**E,G**). (**H**–**J**) This is the parafoveal cystoid space/diffuse fluid type. In Segment 1, parafoveal cystoid spaces can be observed at both Henle’s fiber layer and INL (H and I). Diffuse fluid can be observed at Segment 2 (**H**,**J**). (**K**–**M**) This is the diffuse fluid/diffuse fluid type. Diffuse fluid can be observed at both Segments 1 (**K**,**L**) and 2 (**K**,**M**). (**N**–**P**) This is the diffuse fluid/diffuse fluid with subretinal fluid type. In addition to diffuse fluid at both Segments 1 (**N**,**O**) and 2 (**N,P**), the fluid has accumulated in the subretinal space (asterisks in **N,P**). *IPL* inner plexiform layer, *INL* inner nuclear layer, *OPL* outer plexiform layer, *ONL* outer nuclear layer, *PRS* photoreceptor segments, *RPE* retinal pigment epithelium.

Although there may be exceptions because the number of cases in the present study was not very large, the present study showed that all eyes with DME could be classified into five types based on the localization and area of the accumulated fluid (Figs. 1, 2, 3, 4, 5, 10). This classification is in good agreement with previous reports showing that fluid accumulation in eyes with DME occurs at INL and OPL, including Henle’s fiber layer, the ONL, and the subretinal space, and that the area of fluid accumulation increases as DME progresses^8,9,12,13,18^. The histological characteristics of capillary plexuses in the retina and the results of previous studies using FA and B-scan images indicate that the initial accumulation of fluid predominantly occurs at Henle’s fiber layer because the fluid flows directly from the leaky microaneurysm of the deep capillary plexus (DCP) to Henle’s fiber layer, which shows lower resistance relative to that shown by the other layers^6–8,8–11,13,35,36^. When leakage from the intermediate capillary plexus and DCP increases, the fluid is primarily trapped between the IPL and the synaptic portion of the OPL, i.e., the INL, because both the IPL and the synaptic portion of the OPL act as barriers to fluid movement^3,13,37^. Fluid accumulation at the outer retinal layer has been reported to occur later than fluid accumulation at the inner retinal layer during the course of DME progression^3,38^. Fluid accumulation at the ONL is caused by the influx of fluid from the INL^12,13,37^, and this phenomenon in the subretinal space is thought to be caused by inefficient fluid removal by the dysfunctional RPE or fluid movement from the ONL through a weakened and permeable ELM^3,39–41^. Although the present study was not longitudinal, our classification appears to be in good agreement with the process of DME progression (Fig. 10). Future longitudinal studies are needed to clarify the process of DME progression.

This study has several important limitations. First, it was retrospective. Second, the number of cases was relatively small. Prospective examination of a larger number of cases is necessary for improving the accuracy of the proposed en face image-based classification. Third, although we automatically and objectively delineated Segment 1 and Segment 2 using the SS-OCT Triton (Topcon Corporation) device and the accompanying software, we cannot say whether other OCT machines will be similarly successful. Particularly, when delineating the IPL/INL for the segmentation of Segment 1, the boundary was delineated according to Triton’s original algorithm, which has not been released to the public. Furthermore, the en face images of Segment 2 may have shown outward extension of the fluid in Henle's fiber layer or shadowing by the hyperreflective lesion at the inner retina. Fourth, only eyes with treatment-naïve DME were included, and eyes with end-stage DME, such as those with atrophic diabetic maculopathy, were not included. Fifth, the detection power for incomplete PVD and EZ disruption was limited because the judgment was only based on horizontal and vertical B-scan images centered at the fovea. Finally, the present study did not examine the effect of retinal ischemia on visual functions in DME^38^. Future studies on the relationships between retinal ischemia and visual function or the classification of DME are needed.

In conclusion, we proposed a classification for DME using en face imaging, with focus on both the area and localization of the fluid, and showed that the presence of fluid at Segment 2 was significantly associated with visual acuity, EZ continuity, and CST. Because it has been shown that fluid at the outer retinal layer is less responsive to anti-VEGF drugs or steroids and intractable^5,39^, the presence of fluid in Segment 2 should be precisely determined using en face imaging.

## Methods

### Study design and subjects

This retrospective, cross-sectional study included 128 eyes of 93 Japanese patients diagnosed with center-involving DME from January 2018 to April 2020. Only eyes that had never received anti-VEGF drugs, steroids, or vitrectomy were included. Eyes with a history of laser treatment for peripheral retinal nonperfusion were also included. The exclusion criteria were as follows: a history of macular diseases that cause edema, such as retinal vein occlusion; cataract with a density of grade 3 or higher according to the Emery–Little classification, high myopia (<− 6 D of spherical correction)^40^, and glaucoma. All the investigative procedures conformed to the tenets of the Declaration of Helsinki. The study was approved by the Ethics Committee of Okayama University Hospital, Okayama, Japan. Informed consent was obtained from all participants.

### Ophthalmological examinations

All patients underwent comprehensive ophthalmological examinations, including BCVA testing with refraction using a 5-m Landolt C acuity chart, indirect and contact lens slit-lamp biomicroscopy, and SS-OCT.

### Swept-source optical coherence tomography

For construction of B-scan and en face images, 3D volume data of the retina were obtained over a 6 × 6-mm area consisting of A-scans with 512 lines × 256 lines. The en face images were analyzed using IMAGEnet6 (Version 1.22 software, Topcon Corporation, Tokyo, Japan), which was installed on Triton. On the basis of the retinal layer boundary information, IMAGEnet6 can align the 3D OCT volume data along a specific retinal layer boundary and generate an en face image at an arbitrary depth.

### Classification of diabetic macular edema based on en face images

DME was classified on the basis of the en face images. To construct the en face images, we segmented the retina into two segments using the IMAGEnet 6 software: Segment 1, which primarily comprised the INL and OPL, including Henle’s fiber layer; and Segment 2, which primarily comprised the ONL. For further segmentation of Segment 1, we selected the IPL/INL as the reference plane for flattening the retina and set the scan depth to 50 μm because the thickness of the INL is reportedly 33–40 μm^41–43^, and we considered it necessary to ensure that the scan depth was a little greater than the thickness of the INL for visualization of the OPL, including Henle’s fiber layer, in addition to the INL (Supplementary Figure 1). For further segmentation of Segment 2, we selected the RPE as the reference plane for flattening the retina. Because the thickness from the RPE to the ELM is reportedly 60–80 μm, and the thickness of the ONL is 65–70 μm^41–43^, we moved the segmentation line of the RPE upward by 70 μm from the RPE to the ONL and set this as the inferior boundary of Segment 2. Then, the scan depth was set to 50 μm for ONL visualization (Supplementary Figure 1). The fluid at Segment 1 was classified according to its extent. Specifically, when the fluid was localized within the foveal area on the ETDRS map (circle with a 1-mm diameter centered at the fovea), it was defined as FC (Fig. 1). When the area of fluid was larger than the foveal area but within the parafoveal area on the ETDRS map (circle with a 3-mm diameter centered at the fovea), it was defined as PC (Figs. 2, 3). When the area of fluid was larger than the parafoveal area, it was defined as DF (Figs. 4, 5). With regard to the fluid at Segment 2, we examined both the presence of fluid and SF. DME was classified by three vitreoretinal specialists (A.F., Y.K., and Y.M.).

### Evaluation of incomplete posterior vitreous detachment and disruption of the ellipsoid zone

Incomplete PVD and EZ disruption were evaluated using both horizontal and vertical B-scan images (scan length 12 mm) centered at the fovea. We considered incomplete PVD to be present when the posterior hyaloid detached from the retina partially or completely but remained attached to the optic disc^26^. EZ disruption was evaluated within a 3-mm width centered at the fovea and judged when disruption was observed on either horizontal or vertical B-scan images^44^.

### Measurement of the central subfield thickness

CST was defined as the average thickness of the retina within the foveal area on the ETDRS map. It was automatically measured with IMAGEnet6^45–47^.

### Detection of epiretinal membrane and measurement of the maximum depth of retinal folds

To visualize the en face image of the retinal surface, we flattened the 3D OCT volume scan data at the level of ILM. Using the en face image of the retinal surface, we investigated the presence of ERM, defined as irregular and hyperreflective membrane-like structures^17^. For eyes with ERM, we measured the MDRF according to previous reports^16,17^. Briefly, we visualized the black lines corresponding to the retinal folds due to retinal traction by ERM on the en face image below the ILM level. Then, we measured the depth from the ILM just before the deepest retinal fold disappeared.

### Statistical analysis

All data are expressed as the mean ± standard deviation. BCVAs were recorded as decimal values and converted to logarithm of the minimal angle of resolution units for statistical analysis. All statistical analyses were performed using SPSS version 22.0 (IBM Corp, Armonk, NY, USA). The χ^2^ test of independence was used to analyze the relationships of the en face image-based classification of DME with the number of cases, sex, incomplete PVD, EZ continuity, and incidence of ERM, while one-way analysis of variance was used to analyze the relationships of the classification with age, HbA1c, CST, and MDRF. The Kruskal–Wallis test was used to determine the relationship between the classification and BCVA. With regard to fluid at Segment 2, its relationships with BCVA and CST were analyzed using Student’s t-test, while its relationships with EZ continuity and the incidence of ERM were analyzed using the χ^2^ test. To clarify the factors associated with EZ disruption, we performed logistic regression analysis with classification of DME, BCVA, and CST as independent variables. A *P* value of < 0.05 was considered statistically significant.

## Supplementary Information

Supplementary Figure S1.

Supplementary Figure Legend.
