# Supplementary material for: En face image-based classification of diabetic macular edema using swept source optical coherence tomography
Source: Sci Rep. 2021 Apr 7;11:7665. doi: 10.1038/s41598-021-87440-3 (PMC8026626; doi:10.1038/s41598-021-87440-3)

En Face Image-Based Classification of Diabetic Macular Edema Using Swept Source Optical Coherence Tomography

Atsushi Fujiwara,[1,2] Yuki Kanzaki,[1] Shuhei Kimura,[1] Mio Hosokawa,[1] Yusuke Shiode,[1] Shinichiro Doi,[1] Kosuke Takahashi,[1] Ryo Matoba,[1] Yuki Morizane[1,*]

Institutional Affiliation:

[1] Department of Ophthalmology, Okayama University Graduate School of Medicine, Dentistry and Pharmaceutical Sciences, Okayama 700-8558, Japan
[2] Department of Orthoptics, Faculty of Rehabilitation, Kawasaki University of Medical Welfare, Okayama 701-0193, Japan

*moriza-y@okayama-u.ac.jp

Suppl fig. 1

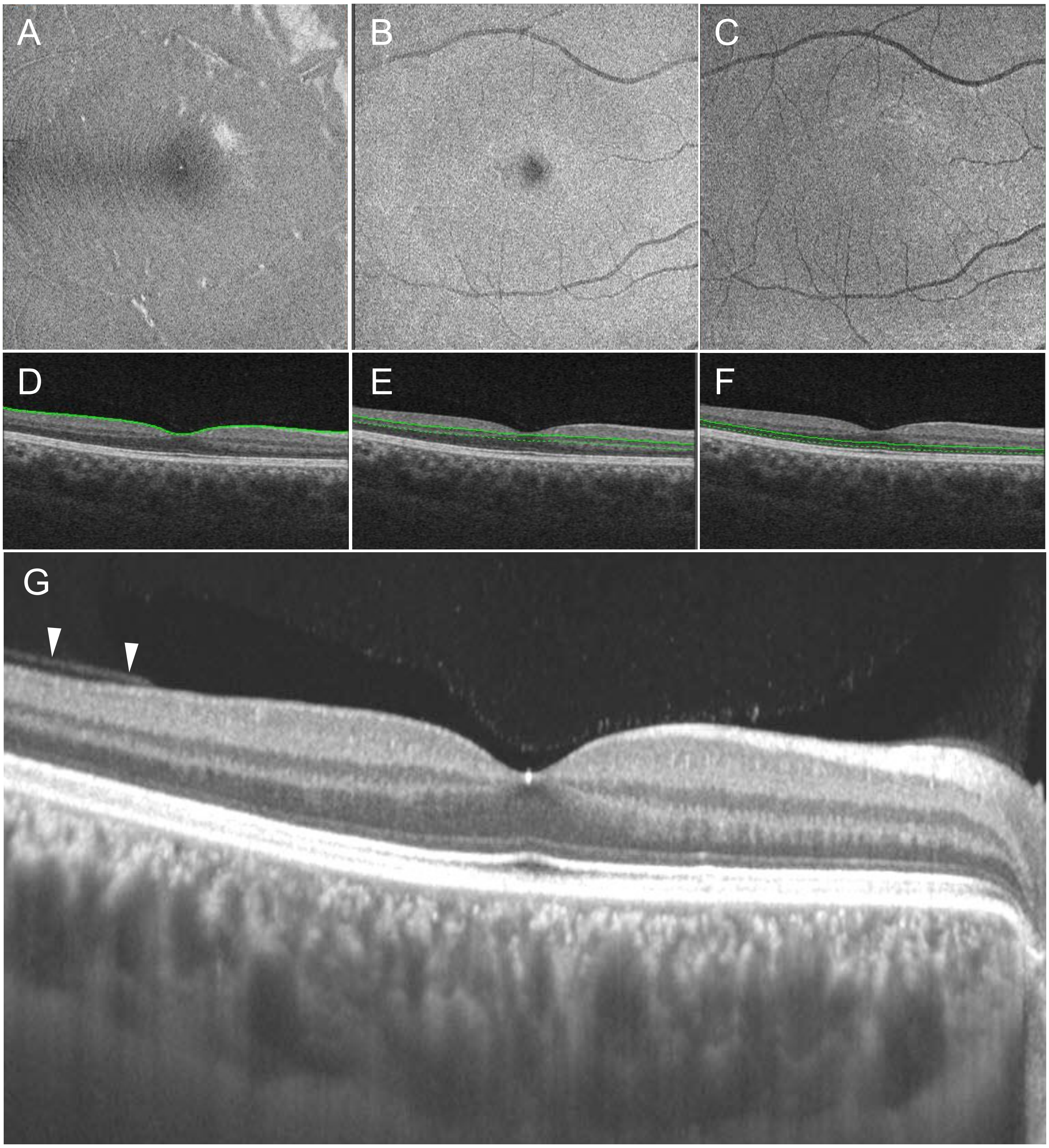

Supplement: Supplementary file 1 — Supplementary Figure S1. [file 41598_2021_87440_MOESM1_ESM.pdf]
