# Supplementary material for: En face image-based classification of diabetic macular edema using swept source optical coherence tomography
Source: Sci Rep. 2021 Apr 7;11:7665. doi: 10.1038/s41598-021-87440-3 (PMC8026626; doi:10.1038/s41598-021-87440-3)
Supplement: Supplementary file 2 — Supplementary Figure Legend. [file 41598_2021_87440_MOESM2_ESM.docx]

Supplementary Figure 1. Representative optical coherence tomography images of a healthy retina (A-C) En face images of the retinal surface (A), Segment 1 (B), and Segment 2 (C) are presented. The surface of the retina is smooth, and there is no epiretinal membrane (A). There are no fluid at both Segments 1 and 2 (B-C). (D-F) B-scan images and the green lines show the locations at which the en face images of the retinal surface (D), Segment 1 (E), and Segment 2 (F) were generated. The scan depth, indicated by the distance between the green line and green dotted line, was set to 0 μm for the en face image of the retinal surface (D) and 50 μm for the en face images of both Segments 1 and 2 (E and F, respectively). (G) A horizontal B-scan image centered at the fovea is presented. The arrowheads in G show that the posterior hyaloid is partially detached from the retina.
